# R Shiny App for the Automated Deconvolution of NMR Spectra to Quantify the Solid-State Forms of Pharmaceutical Mixtures

**DOI:** 10.3390/metabo12121248

**Published:** 2022-12-10

**Authors:** Piotr Prostko, Jeroen Pikkemaat, Philipp Selter, Michail Lukaschek, Rainer Wechselberger, Tatsiana Khamiakova, Dirk Valkenborg

**Affiliations:** 1 Data Science Institute, UHasselt—Hasselt University, Agoralaan 1, BE 3590 Diepenbeek, Belgium; 2 Interuniversity Institute for Biostatistics and Statistical Bioinformatics (I-BioStat), Agoralaan 1, BE 3590 Diepenbeek, Belgium; 3 Janssen Pharmaceutica, Department of Analytical Development, Turnhoutseweg 30, BE 2340 Beerse, Belgium; 4 Janssen Pharmaceutica, Manufacturing and Applied Statistics, Turnhoutseweg 30, BE 2340 Beerse, Belgium

**Keywords:** solid-state NMR, deconvolution, quantification, pharmaceutical development, quality control, software, R Shiny, non-linear optimisation

## Abstract

Bioavailability and chemical stability are important characteristics of drug products that are strongly affected by the solid-state structure of the active pharmaceutical ingredient (API). In pharmaceutical development and quality control activities, solid-state NMR (ssNMR) has proved to be an excellent tool for the detection and accurate quantification of undesired solid-state forms. To obtain correct quantitative outcomes, the resulting spectrum of an analytical sample should be deconvoluted into the individual spectra of the pure components. However, the ssNMR deconvolution is particularly challenging due to the following: the relatively large line widths that may lead to severe peak overlap, multiple spinning sidebands as a result of applying Magic Angle Spinning (MAS), and highly irregular peak shapes commonly observed in mixture spectra. To address these challenges, we created a tailored and automated deconvolution approach of ssNMR mixture spectra that involves a linear combination modelling (LCM) of previously acquired reference spectra of pure solid-state components. For optimal model performance, the template and mixture spectra should be acquired under the same conditions and experimental settings. In addition to the parameters controlling the contributions of the components in the mixture, the proposed model includes terms for spectral processing such as phase correction and horizontal shifting that are all jointly estimated via a non-linear, constrained optimisation algorithm. Finally, our novel procedure has been implemented in a fully functional and user-friendly R Shiny webtool (hence no local R installation required) that offers interactive data visualisations, manual adjustments to the automated deconvolution results, and the traceability and reproducibility of analyses.

## 1. Introduction

**Figure 1:**
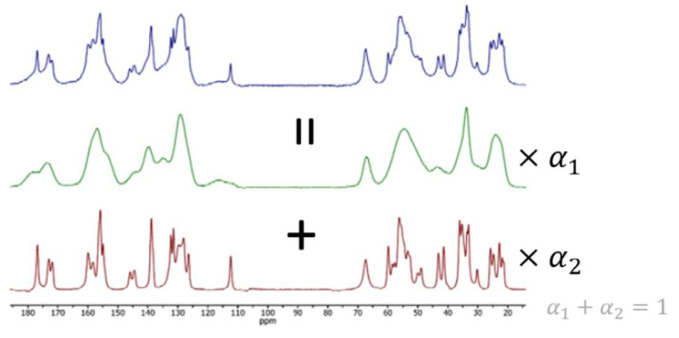
The mixture ssNMR spectrum (in blue) of a compound of interest contains contributions of crystalline (red) and amorphous (green) solid-state forms. The proportions of the two solid-state forms present in the physical mixture can be determined by deconvolution of the mixture spectrum into the two separate pure-component spectra. Only spectrum real parts are shown.

During the Chemistry, Manufacturing, and Controls (CMC) activities of drug development, pharmaceutical scientists aim to establish optimal manufacturing processes and drug formulations, resulting in a safe, stable, and effective product. Bioavailability and chemical stability are important characteristics to ensure the required therapeutic levels and patient safety. Both characteristics are strongly influenced by the solid-state structure of the active pharmaceutical ingredient (API) and, occasionally, also of other constituents of the drug product [1,2]. Therefore, detection and accurate quantification of undesired solid-state forms is essential for pharmaceutical development and quality control. Solid-state NMR (ssNMR) is an excellent tool for this purpose since the shape and position of the ssNMR signal are very sensitive to the solid-state structure. Furthermore, the intensity of a specific ssNMR signal has a linear correlation with the concentration of the corresponding solid form. For these reasons, ssNMR spectra of solid–solid mixtures usually can be accurately approximated by linear combinations of the spectra of the individual components, provided that the ssNMR spectra of the individual components and the mixtures are collected at the same conditions and the same experimental settings. The proportions of these linear combinations have a linear correlation with the proportions of the corresponding solid forms in the mixture. This is exemplified in Figure 1. Thus, to determine the accurate proportions of the individual solid forms from the ssNMR spectrum of a solid–solid mixture, the mixture spectrum first needs to be accurately deconvoluted into the separate spectra of the pure components. In this deconvolution process the reference spectra of the amorphous and crystalline state are used as a template. In the remainder of this manuscript, we term the reference spectra of the individual components as “template spectra”.

A wide range of methods has been developed for NMR signal deconvolution, primarily in the context of metabolite analysis of the human brain in vivo spectra. Methods such as AMARES [3], QUEST [4], AQSES [5], all three available in the jMRUI software suite [6], or TARQUIN [7] perform the modelling of experimental signals in the time domain, while other approaches such as LCModel [8], the method described in [9], GANNET [10], and Osprey [11] operate in the frequency domain. Generally, most of these modelling routines seek to explain an experimental signal with a function (for instance, linear combination) of baseline and experimental or simulated template signals of the pure components that are further modified to account for NMR-specific artefacts, including phase errors, chemical-shift discrepancies, and line-shape distortions. Fitting a mixture of Gaussian or other distribution functions to only selected peaks in a spectrum is another possibility in quantitative NMR spectroscopy [10,12]. Exploiting prior knowledge concerning the signal’s characteristics is a common way to aid the non-linear model optimisation towards the correct solution. The previously mentioned methods differ regarding what type of information can be used and how easy it can be incorporated into the modelling process. The prior knowledge typically pertains to the individual signal’s properties such as baseline, peak shape (Gaussian, Lorentzian, or Voigt), and intensity ratios within a peak multiplet, but also the discrepancies between analytical and template signals. 

For several reasons, the methods mentioned above are not well suited to efficiently tackle QC requirements in API manufacturing applications and the general challenges of solid-state NMR data analysis. Firstly, in ssNMR we often seek to quantify different forms of the same compound, which, in combination with the relatively large line widths of the ssNMR signals, gives a high risk of severe overlap in the mixture spectrum. Secondly, the undesired solid-state form, generally the form that needs to be quantified, is usually present at low concentrations. Thirdly, Magic Angle Spinning (MAS) is often employed in ssNMR to enhance spectral resolution. This technique causes the splitting of the original signal into multiple spinning sidebands with varying intensity locations; all the sidebands should be considered by a quantification method. Furthermore, highly irregular peak shapes, typically observed in ssNMR, often preclude accurate parsimonious capturing. The last remark regarding the data characteristics occurring in quality control of APIs is usually a limited number of solid-state forms expected in the final drug product, allowing for building a very targeted, specific, and sensitive deconvolution procedure.

To address these issues and data characteristics elaborated above, we created a dedicated ssNMR mixture spectra deconvolution approach, implemented in R Shiny, that exploits a specific type of prior information available in the context of solid-state polymorph analysis of APIs. The prior knowledge is given by a priori acquired experimental ssNMR data of pure, unmixed samples of solid-state forms of the compound of interest. A linear combination modelling (LCM) of the template spectra is then carried out with a non-linear, constrained optimisation routine such that the sum of squared deviations between the observed and model signals is minimised. For optimal model performance, the template and mixture spectra should be acquired under the same conditions and experimental settings. A full description of the modelling effort and design choices will be published in a separate and more fundamental research article. In this manuscript, we mainly want to draw the readers’ attention to the existence of a fully functional R Shiny app (https://valkenborg-lab.shinyapps.io/ssNMRdeconvolution/, version 1.0.0, accessed on 9 September 2022) for the deconvolution of ssNMR mixture spectra. The function of the app is demonstrated with a limited data set. A validation study with a larger set of experimental data is ongoing.

## 2. Materials and Methods

### 2.1. Algorithm

**Figure 2:**
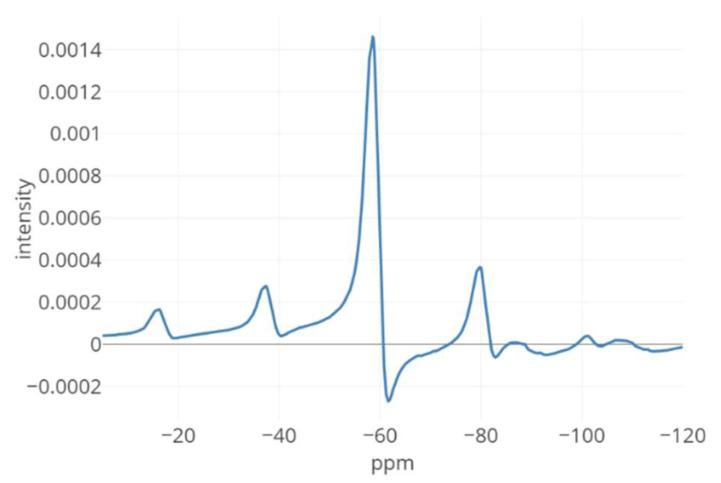
The ssNMR mixture spectrum of a spiked sample of amorphous material containing 3% of a crystalline solid-state form. Among other factors mentioned in the Introduction, the multiple sidebands, broad peaks due to the dominant amorphous component, and processing artifacts such as considerable phase error necessitate a tailored deconvolution approach.

To explain the rationale for constructing our proposed fitting procedure, we first present Figure 2 which shows an example—used throughout the entire manuscript and described more in the Results section—of a solid-state ssNMR measurement of a spiked sample that contains 3% and 97% of crystalline and amorphous solid-state forms of an API, respectively. In that figure, the previously discussed features can instantly be noticed, such as multiple sidebands, broad peaks due to large amorphous material content, and—most strikingly—large phase error.

Phase correction of such broad peaks is challenging for both computers and expert spectroscopists. Manual phase correction by an NMR spectroscopist involves significant time cost and between-spectroscopist variation—an unwanted characteristic of any QC method in the pharmaceutical industry. Therefore, we assume that only the template spectra are manually and carefully phased and then kept fixed within a given quantification project. On the other hand, the analytical mixture spectra are automatically phase corrected by our model. The model also should account for minor errors in chemical shift that may occur while referencing spectra and result in horizontal misalignment between the analysed spectra.

Let $\left(x_{mix,n},\;y_{mix,n}\right),\;\left(x_{form1,n},\;y_{form1,n}\right),\;\left(x_{form2,n},\;y_{form2,n}\right),\;n=1,\ldots ,N$ be the data of the mixture spectrum and the template spectra representing the prior knowledge, respectively. Within this notation, $x_{n}$ and $y_{n}$ are the *n*-th values of chemical shift and intensity (complex number); “form1” and “form2” are the generic terms that correspond to two template spectra (the crystalline and amorphous solid-state forms in this particular application). To account for small differences in the chemical shift between the three spectra, linear interpolation is performed before modelling to assure that the three spectra are at the same ppm scale. The ppm scale of “form2” is used as a reference for this interpolation step. Now, phase and chemical shift errors, together with the proportions of two solid-state forms in the mixture sample, are included in the model that aims at minimising one of two possible loss functions applied to the model residuals in the frequency domain. The two possibilities are quadratic (L2 norm) or least absolute deviation (L1 norm). We chose the quadratic loss function as the default, and it is given by $\overset{N}{\underset{n=1}{\sum }}e_{n}^{2}$ (Residual Sum of Squares, RSS). The residuals are defined as follows:

(1) $$e_{n}={∆}_{mix}\left(Re(\exp\left(i\varphi \left(n\right)\right)\times y_{mix,n}\right))-\alpha_{1}{∆}_{form1}\left(Re\left(y_{form1,n\;}\right)\right)-\alpha_{2}Re\left(y_{form2,n}\right)$$

where Re() denotes the real part of the spectrum and $\alpha_{1},\alpha_{2}$ are the mixing coefficients subject to constraints: $\alpha_{1}$ + $\alpha_{2}=1$ and $0\leq \alpha_{1},\alpha_{2}\leq 1$. The linear interpolation operator is denoted by Δ() and used to move spectra horizontally. Note that while given three spectra at a time, it is sufficient to fix the location of one spectrum and allow the other two to be shifted. Next, the $\varphi \left(n\right)$ factor is responsible for phase correction and is defined as follows:

(2) $$\varphi \left(n\right)=(\varphi_{0}-\frac{\varphi_{1}}{N}n^{*})+\frac{\varphi_{1}}{N}n,\;\;n=1,\ldots ,N$$

In the equation above, $\varphi_{1}\;$ is the first-order phase correction (PH1) coefficient, and $\varphi_{0}$ is the term contributing to the zero-order phase correction (PH0). Moreover, n denotes the index of points in the spectrum, while $n^{*}$ is the index of the pivot point (the point where the first-order phase correction is zero). Due to the inclusion of the pivot point, only the entire term in parentheses in Equation (2) can be interpreted as zero-order phase correction. The RSS is minimised using Sbplx re-implementation of the Subplex algorithm [13], which is a variation of Nelder–Mead’s simplex algorithm. To reiterate, the proposed model is an example of an LCM approach tailored to a specific ssNMR use case (API solid-state form analysis). In this application, the amorphous component in the mixture spectrum is characterised by considerably broad peaks; this property hampers phase correction, which is crucial for accurate deconvolution, hence the decision to jointly optimise the selected processing parameters and the mixing proportions. Additional information on the model implementation in R can be found in Supplementary Materials.

### 2.2. R Shiny Application

**Figure 3:**
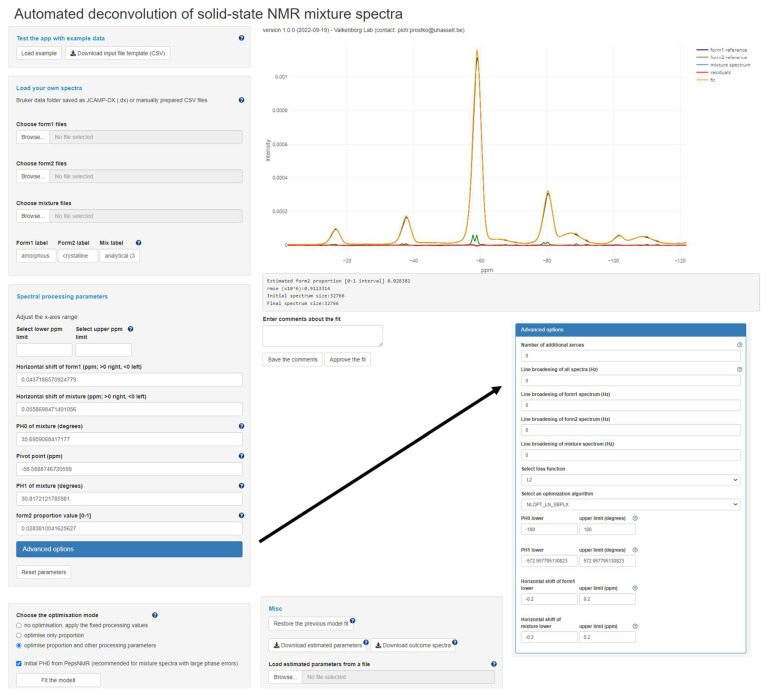
An expanded view of Graphical User Interface. A brief tutorial on how to use the app can be found in Supplementary Materials.

The described deconvolution algorithm has been implemented in a web-based Shiny application available at https://valkenborg-lab.shinyapps.io/ssNMRdeconvolution/ (version 1.0.0, accessed on 9 September 2022), and thus the user can upload the spectral data and interact directly with the app without local R installation. In this section, only main features of the app are discussed; however, a detailed user manual can be found in Supplementary Materials. Figure 3 shows a screenshot of the app’s Graphical User Interface (GUI).

The GUI consists of several numerical and text input fields through which the user provides values affecting spectral processing and deconvolution, action buttons that assist in data loading and trigger certain actions such as model fitting or results downloading, and an interactive graph showing the mixture and template spectra together with the fitted spectrum and the residual line. Note that the graph is a truly dynamic and fully functional visualisation interface that allows a user to interact with different uploaded ssNMR spectra. The right-hand side of the figure displays a legend and zoom functionality. By clicking on the legend, a user can turn on/off certain spectra or results of the fitting procedure. On the left-hand side, several functionalities including model fitting are located, and those will be further explained in the user manual available in Supplementary Materials.

The typical session starts with loading spectral data in the form of either a JCAMP-DX file or two CSV files. Do consult Supplementary Materials to learn how to prepare the input data. Furthermore, three distinct ways of working with the app have been envisioned and implemented:

- “no optimisation, apply the fixed processing values”: No optimisation is performed; the manually specified values in the input fields corresponding to ${∆}_{mix},{∆}_{form1},\;\varphi_{0},\varphi_{1},\;\alpha_{2}$ (note that $\alpha_{1}=1-\alpha_{2}$) are directly applied to process (according to Equation (1)) and visualise the spectra.
- “optimise only proportion”: The same workflow as above, except that now the $\alpha_{2}$ parameter is estimated, offering a compromise between fully manual and fully automated processing and deconvolution.
- “optimise proportion and other processing parameters”: This entails the fully automated optimisation of the five model parameters.

The loss function minimised by the optimisation algorithm app can be switched by the user from quadratic deviation (L2, default choice) to the least absolute deviation (L1).

Note that it is possible to run the same estimation form multiple times and/or switch from one to another. This flexibility grants greater control over the deconvolution process because the user can, for instance, run the fully automated optimisation, assess the model fit, and then manually adjust the obtained solution via the input fields to try to improve the fit. Every user’s action is recorded in a downloadable CSV file, allowing to pause and restart the analysis at a different occasion, thereby promoting traceability and reproducibility.

## 3. Results

To develop and test our ssNMR deconvolution approach based on LCM, we used in-house-prepared tablets mimicking as closely as possible the intended commercial pharmaceutical product. These tablets contain 0%, 3%, 10%, 20%, and 100% crystalline Apalutamide in otherwise amorphous solid dispersion Apalutamide, granulate Zytiga, and external phase excipients. The total weight percent of Apalutamide in all tablets is 6%. Details on sample preparation and NMR acquisition are provided in Supplementary Materials.

**Figure 4:**
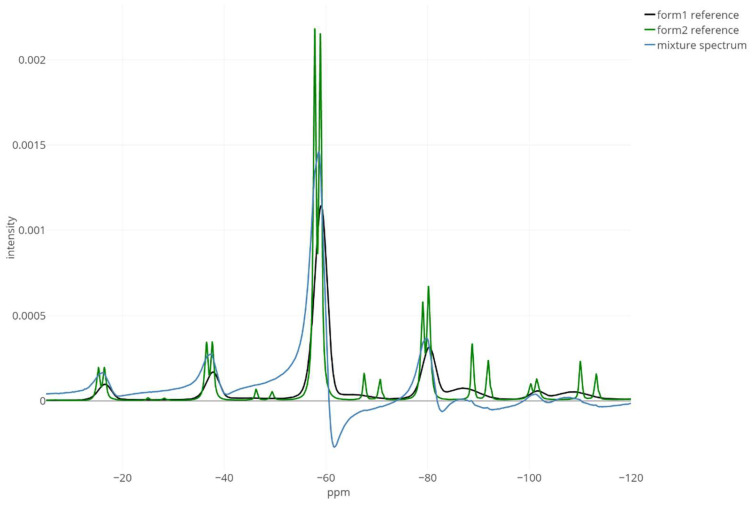
The before-deconvolution visualisation of the 3%-crystalline mixture spectrum (in blue) together with the 100% pure amorphous (“form1”, the black line) and 100% pure crystalline (“form2”, the green line) templates. The discussed processing and deconvolution challenges are now clearly visible: severe peak overlap, large phase error of the mixture, and broad peaks of the amorphous component. Only spectrum real parts are shown. The x-axis range was limited to −5 and −120 ppm for better visibility.

We analysed one spectrum of a 3%-crystalline tablet to demonstrate our novel Shiny app. To make the data more challenging for the developed deconvolution method, the manual zero-order phase correction described in the Supplementary Materials was deliberately reverted. Figure 4 presents the analytical spectrum and two templates, where it can be immediately noticed that the mixture spectrum contains a large phase error and perhaps a slight horizontal misalignment between the three signals.

**Figure 5:**
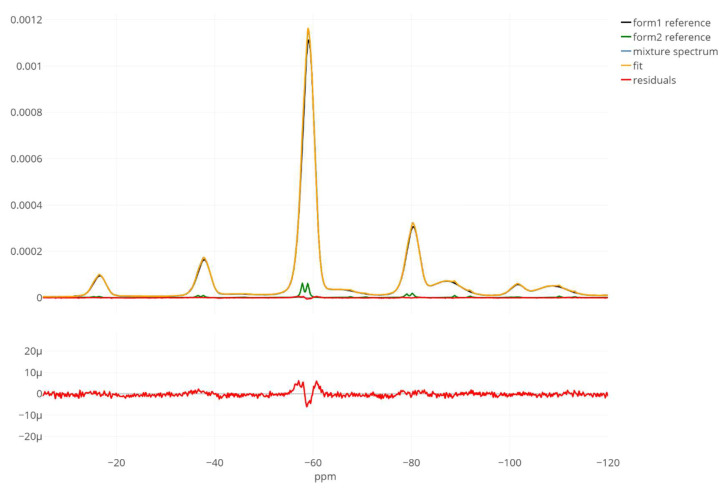
A satisfactory model fit obtained with the automated “proportion and processing parameters” mode. The true proportion of the crystalline spectrum (“form2 reference”, the green line) in the mixture (the blue line) spectrum is 3%, whereas the model estimate points at 2.84%, yielding a −5.5 relative error (in percentage points). The bottom panel is a zoomed view on the residual line alone that confirms the good quality of the found solution. Only spectrum real parts are shown. The x-axis range was limited to −5 and −120 ppm for better visibility.

Running the “proportion and processing parameters” estimation mode resulted in an estimate of the crystalline proportion equal to 2.84% (and thus the relative error of −5.5 percentage point) and a good model fit as judged by a small and randomly fluctuating residual line (Figure 5). The phase error was successfully removed from the mixture spectrum since the peaks are noticeably more symmetrical and take positive values. In this case, the obtained results did not necessitate manual adjustments nor further model re-estimation using various GUI features.

## 4. Discussion

To bridge the gap between general-purpose quantitation software for in vivo NMR spectroscopy and ssNMR analysis of APIs, we created a linear combination modelling method for spectral deconvolution allowing the efficient use of information regarding signal properties represented by previously acquired experimental template spectra. This choice has substantially limited the number of otherwise needed parameters to model the erratic, wide peak shapes and spinning sidebands in ssNMR spectra. Such a reduction in the parameter space is especially appreciated in non-linear optimisation problems. Overall, we demonstrated that the method works well on one ssNMR spectrum of a synthesised drug tablet. A validation study involving a larger set of experimental spectra will be forthcoming. While this application note focusses on pharmaceutical APIs, the described method is obviously also suitable for mixtures of solid forms of other pure compounds. For instance, the solid form constitution of excipients and its stability can be analysed in the raw materials or even in the finished drug product. Moreover, the flexible design of the R Shiny application, with several options for manual results adjustments, should alleviate the burden associated with transitioning from a proprietary software. As in many non-linear models, our approach might also be sensitive to the parameter starting values. However, we supply sensible values for the horizontal shift coefficients (zeros, as it is expected that the input spectra should be provided already reasonably well aligned), the “form2” proportion (zero, as we assume that the “form2” represents the unwanted and less abundant solid-state form), and the zero-order phase, which can be automatically initialised based on a method that maximises the positiveness of the real part of the spectrum [14]. Thus, only the first-order phase correction term is left out and could be varied to see its impact on the model fit. Nevertheless, the validity of starting values and the resulting parameter estimates and how these could be improved can be inferred by looking at the residual, fitted, and individual spectral lines on the interactive graph. Even though using experimental template spectra allowed for specifying a parsimonious model, differences in handling analytical and reference samples that cause discrepancies in the corresponding spectra may affect the quality of the final solution. Further research will be devoted to providing the standard errors of the model parameters because, at this moment, the uncertainty around the mixing proportions can only be assessed based on a calibration study. We will also attempt to expand the proposed methodology to accommodate for the multiple template scenarios where more than one crystalline or amorphous solid-state form is to be quantified.

## Data Availability

The data used in this research are available in the Shiny app (https://valkenborg-lab.shinyapps.io/ssNMRdeconvolution/, version 1.0.0 accessed on 9 September 2022) via the Load example button.

## Supplementary Materials

The following supporting information can be downloaded at: https://www.mdpi.com/article/10.3390/metabo12121248/s1, Figure S1: Annotated GUI of the Shiny app; Figure S2: Exporting spectra from TopSpin in a JCAMP-DX format; Figure S3: Input file data structure for loading CSVs; Figure S4: Three optimisation modes; Figure S5: Spectral processing parameters; Figure S6: Advanced options for spectral processing and optimisation; Figure S7: Interactive visualisation; Figure S8: Explanation of the toolbar in the Interactive visualisation; Figure S9: Other useful buttons in GUI; Figure S10: Description the CSV file storing optimisation results; Figure S11: Selected model fit information; References are cited in Supplementary Materials [15,16,17]
