# Supplementary material for: R Shiny App for the Automated Deconvolution of NMR Spectra to Quantify the Solid-State Forms of Pharmaceutical Mixtures
Source: Metabolites. 2022 Dec 10;12(12):1248. doi: 10.3390/metabo12121248 (PMC9786300; doi:10.3390/metabo12121248)
Supplement: Supplementary file 1 [file metabolites-12-01248-s001.zip › metabolites-1975964-supplementary.pdf]

R Shiny app for the automated deconvolution of NMR spectra to quantify the solid-state forms of pharmaceutical mixtures

Piotr Prostko [1,2], Jeroen Pikkemaat [3], Philipp Selter [3], Michail Lukaschek [3], Rainer Wechselberger [3], Tatsiana Khamiakova [4] and Dirk Valkenborg [1,2,*]

[1] Data Science Institute, UHasselt—Hasselt University, Agoralaan 1, BE 3590 Diepenbeek, Belgium
[2] Interuniversity Institute for Biostatistics and Statistical Bioinformatics (I-BioStat), Agoralaan 1, BE 3590 Diepenbeek, Belgium
[3] Janssen Pharmaceutica, Department of Analytical Development, Turnhoutseweg 30, BE 2340 Beerse, Belgium
[4] Janssen Pharmaceutica, Manufacturing and Applied Statistics, Turnhoutseweg 30, BE 2340 Beerse, Belgium
* Correspondence: dirk.valkenborg@uhasselt.be

Keywords: solid-state NMR; deconvolution; quantification; pharmaceutical development; quality control; software; R Shiny; non-linear optimization

---

Supplementary Materials

Table of Content

- Figure S1 Annotated GUI of the Shiny app
- Figure S2 Exporting spectra from TopSpin in a JCAMP-DX format
- Figure S3 Input file data structure for loading CSVs
- Figure S4 Three optimisation modes
- Figure S5 Spectral processing parameters
- Figure S6 Advanced options for spectral processing and optimisation
- Figure S7 Interactive visualisation
- Figure S8 Explanation of the toolbar in the Interactive visualisation
- Figure S9 Other useful buttons in GUI
- Figure S10 Description the CSV file storing optimisation results
- Figure S11 Selected model fit information

Tablet composition and manufacturing
Calibration reference tablets were prepared containing 0%, 3%, 10%, 20%, and 100% crystalline Apalutamide (molar percentage of crystalline relative to total Apalutamide API) and otherwise mimicking as closely as possible the intended commercial pharmaceutical product. The total weight percentage of Apalutamide in all calibration tablets was 6%. For this purpose, appropriate amounts of the Apalutamide amorphous solid dispersion (25% w/w Apalutamide in hydroxypropyl methylcellulose), crystalline Apalutamide, granulate Zytiga, and excipients were weighed with 1% relative accuracy, sieved and brought in a glass bottle, and blended. Each blend was produced at 300 g batch size. Approximately 20 tablets of each crystalline percentage were manufactured on a single-punch tablet press with compression forces similar to the intended commercial product. Tablet weights were measured manually for all tablets. Core tablets were film coated.

Solid-state NMR spectroscopy

All NMR spectra were acquired on a Bruker AVANCE-III spectrometer equipped with a 8.9 mm bore-size 11.7 Tesla magnet, a 4mm HFXY CP-MAS probe, and a Bruker BCU-II cooling unit. Solid-state MAS ^{19}F NMR spectra intended for quantitation (10 kHz MAS rotation, 90° flip angle, 2048 complex data points, 3.333 µs dwell time, Bruker "baseopt" digitisation mode, 20 s relaxation delay, 5120 signal accumulations) were acquired using high-power ^{1}H decoupling (Bruker "hpdec" pulse sequence, "spinal64" composite-pulse decoupling sequence, 75 kHz decoupling power). All NMR spectra were acquired at 293 K (meter reading). Post-acquisition spectral processing was performed in a fully automated fashion using a dedicated script in Bruker "xau" macro language consisting of (1) multiplication with a 20 Hz exponential function, (2) zero-filling to 32768 complex data points, (3) complex Fourier transform, and (4) zero-order phase correction. Solid-state NMR spectra intended for the determination of the ^{19}F longitudinal relaxation time were acquired using a saturation-recovery pulse sequence (Bruker "satrect1") with 16 saturation-recovery delays in the range between 0.01 and 20.00 s. All other parameters were set to the same values as used for the acquisition of the spectra intended for quantitation. Acquisition and processing parameters, as well as post-acquisition spectral processing, were controlled from within Bruker's proprietary software TopSpin version 3.5 patch level 7. The recorded digitised data adhere to the standard Bruker data storage format.

Technical details

Here we discuss various technical comments on the proposed modelling approach and its implementation in R in the form of a numbered list.

1. Our template-based method requires all data collection at the same conditions and experimental settings. Any significant differences may be reflected in the resulting spectra and a worse model fit. The use of such controlled experimental template spectra is an essential feature, that is intended to improve accuracy and robustness of the quantitation. We anticipate that such template spectra can be rather easily provided for the majority of the quantitative ssNMR applications in pharma and probably also in many other industries and material sciences.
2. Our fitting routine is suited for analysing 1D ssNMR spectra and is not nucleus-specific (so far the app has been used and tested on ^{19}F and ^{13}C spectra).
3. The model fitting procedure has been implemented in R programming language [1] with an R interface [2] to the Nlopt optimisation library [3]. This library enables solving non-linear problems with one of a wide selection of specialised optimisation algorithms. We chose "Sbplx", a local, gradient-free, iterative procedure for finding a local optimum of a non-linear function. The user in app's GUI can change the algorithm. Moreover, the optimisation routine requires providing:
    a. Starting values: the $\Delta_{mix}, \Delta_{form1}, \varphi_0, \varphi_1, \alpha_2$ parameters are all initialized with a zero value. For φ_0, there is option to use the PepsNMR R package [4] to get the starting value. The initialization of φ_0 is then obtained based on minimising the ratio between the sum of squares of positive intensities and the sum of squares of all intensities in the spectrum. Note that the starting values can be manually changed by the user in GUI.
    b. Parameter constraints: For $\varphi_0, \varphi_1, \alpha_2$ the constraints are as follows: for [-180°, 180°] for φ_0, [-573°, 573°] for φ_1, and [0, 1] for α_2. For $\Delta_{mix}, \Delta_{form1}$, the limits are automatically set to -/+ the half of the ppm axis range of all three spectra. We opted for such broad limits to avoid getting estimates exactly at the boundaries of parameter space. Note that all constraints can be manually changed by the user in GUI.

c. Stopping criteria: the calculation stops after achieving 1500 iterations when for every optimized parameter, the absolute or relative change in values between consecutive iterations is less than 10^{-6}.

4. The model equation in the main text indicates the order of processing operations, which is: phase correction, followed by taking the real part, followed by horizontal shifting. What is not mentioned is that in the next step, all three spectra are divided by their individual total intensity so that the α_1, α_2 coefficients can be restricted to the [0, 1] interval.

Description of Graphical User Interface (GUI)

Abbreviations

acq man	TopSpin Acquisition Commands and Parameters (version 011)
Form2	Form2 reference spectrum
PH0	zero-order phase correction
PH1	first-order phase correction
proc man	TopSpin Processing Commands and Parameters User Manual (version 005)

This section explains in detail how to use the app's GUI and is offered towards Windows users. This short guide is applicable to the version 1.0.0 (2022-09-19) of the app. The overview of GUI is presented in Figure S1.

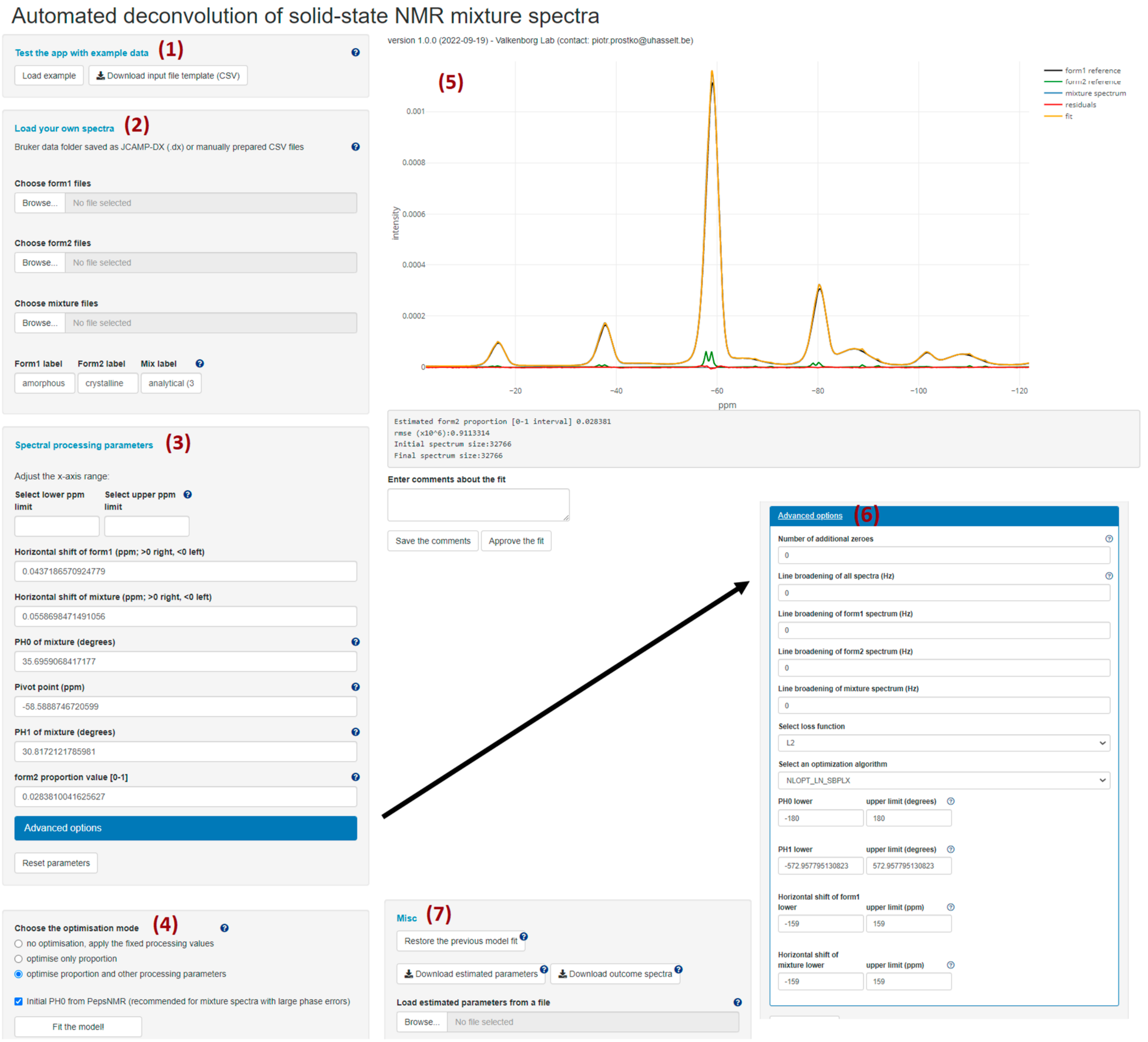

Figure S1 An annotated GUI of the app; each numbered panel is discussed in the following subsections.

1.1 Test the app with example data (1)

The most painless way to familiarise yourself with the app is to read this user manual and immediately test in practice the described functionalities. For precisely this purpose, we embedded in the app a set of three example spectra (one mixture and two reference samples). Clicking on the *Load example* button activates the underlying dataset and promptly triggers an interactive visualisation. That example spectra become available to all functionalities so that the user is free to explore and learn the app's behaviour. *Download input file template* offers the download of the ZIP file containing six CSV files (two CSV files per spectrum) with the data mentioned above. It is advised to open the data of at least one spectrum to understand the required input data structure for the CSV file upload.

1.2 Load your own spectra (2)

We support two "semi-vendor-independent" ways of loading input data.

The first one is suited for loading JCAMP-DX files (the .dx extension) that have been generated by exporting Bruker spectra from TopSpin. As shown in Figure S2 this can be achieved by opening a spectrum in TopSpin, clicking on the three white horizontal bars icon in the top-left corner of the window (or simply pressing "CTRL + S"), selecting "Save data set in a JCAMP-DX file" with default

options. For more information look for "tojdx" procedure in the "proc man". At the moment of writing this document it is unknown if spectra acquired using spectrometers of other vendors and saved as a JCAMP-DX file can be uploaded to the app.

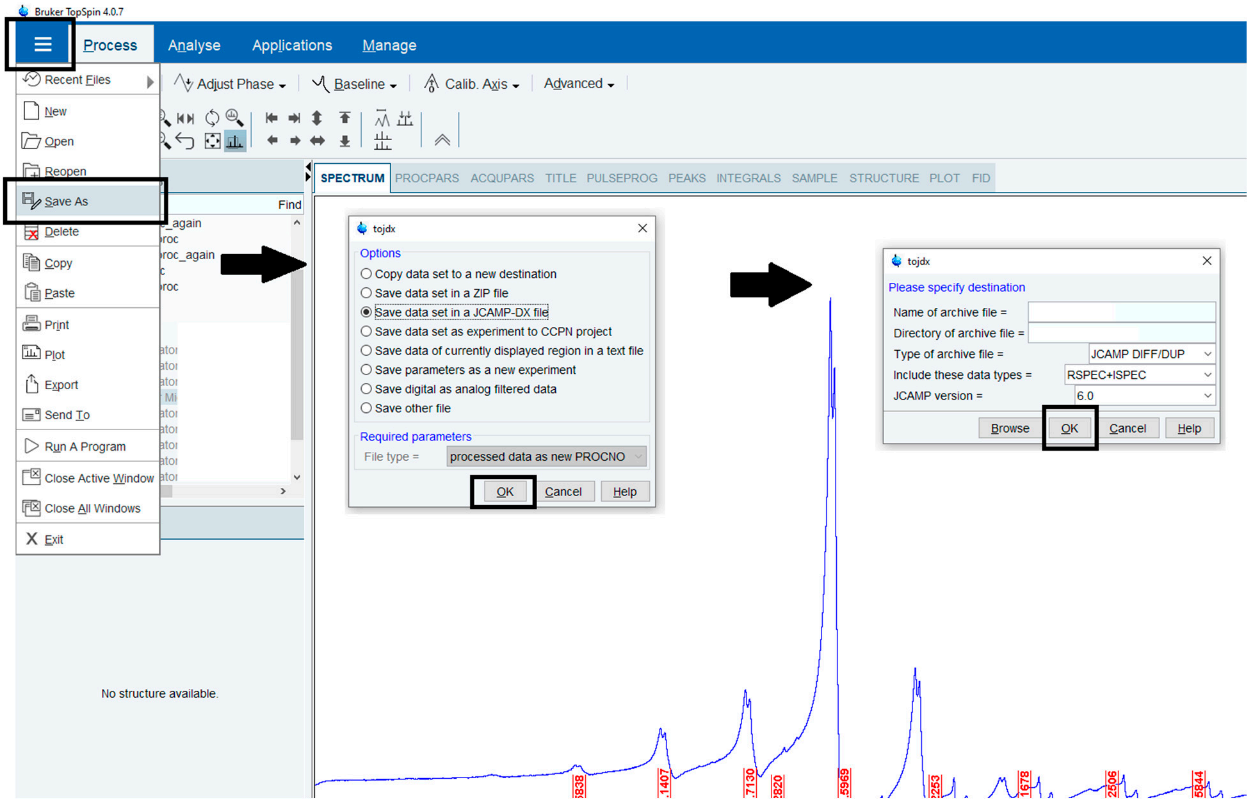

Figure S2 Exporting spectra from TopSpin in a JCAMP-DX format.

The second allowed data format is CSV (two files per spectrum, see Figure S3). The CSV file names must include the following suffixes:

- xx_intensity.csv: This file should include only one column with a header named "intensity". The values are complex numbers corresponding to the loaded spectrum (i.e. signal in the frequency domain).
- xx_params.csv: This file stores selected acquisition and processing parameter values needed for computing the chemical shift values (x-axis) and performing apodisation. The file consists of four columns and two rows (of which one is the column header). Although here we offer an open format for loading the data, these acquisition and processing parameters should resemble the meaning stipulated in "TopSpin Acquisition Commands and Parameters (version 011)" [abbreviation: acq man] and "TopSpin Processing Commands and Parameters User Manual (version 005)" [abbreviation: proc man], and briefly discussed below:
  - **OFFSET**: the ppm value of the first data point of the spectrum; described in "proc man"; the value resides in the "procs" Bruker's file.
  - **SW_p**: spectral width of the processed data (in Hz); described in "proc man"; the value resides in the "procs" Bruker's file.
  - **SF**: spectral reference frequency (or spectrometer frequency; in MHz); described in "proc man"; the value resides in the "procs" Bruker's file.
  - **GRPDLY** (optional): described in "acq man"; the value resides in the "acqus" Bruker's file. In general, this parameter corrects for the first order phase error introduced by the Bruker digital filter [4]. In our implementation, this parameter only

affects the outcome of apodisation. It should be set to zero if no information is available.

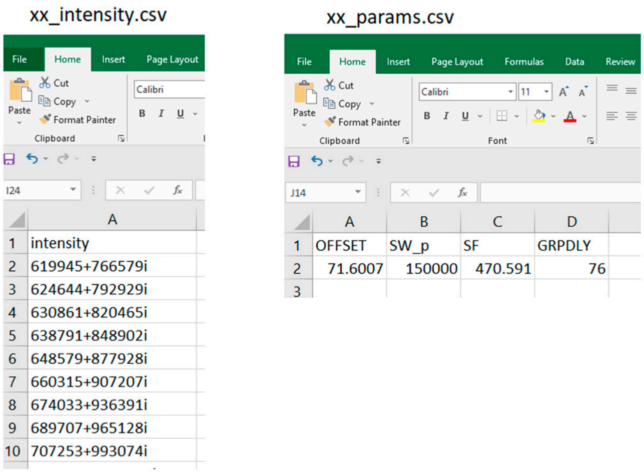

Figure S3 CSV input file data structure.

Either JCAMP-DX or CSV, the input files have to be uploaded from a local computer by clicking on the *Browse* buttons. Finally, after a successful data upload, an interactive graphics of all three initial spectra pops up.

1.3 Choose the optimisation mode (4)

The user can carry out one of three distinct analysis workflows, namely: *no optimisation, apply the fixed processing values*, *optimise only proportion*, and *optimise proportion and other processing parameters* (Figure S4). To execute a preferred analysis, click on the appropriate radio button and accept the choice by clicking *Fit the model!*. Depending on the selected estimation mode, the calculations may take up to several minutes, and while doing so, the “Model fitting is running” message is displayed in the bottom-right corner. Afterwards, the model fitting outcomes can be assessed based on the model fit graph (section *Fit visualisation (5)*).

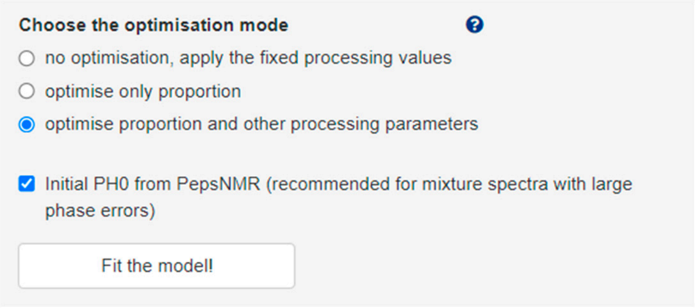

Figure S4 Three different optimisation modes offer greater flexibility for the user (e.g., manual adjustment of the results obtained with full and automated optimisation).

In each workflow, the model parameters from the main article are treated differently:

- ***no optimisation, apply the fixed processing values***: As shown already in Figure S1, under *Spectral processing parameters* section of the GUI, the user can manually provide the values of: ppm range of the analysis, the horizontal spectral shifts; PH0, a pivot point (a point on the x-axis where PH1 correction is zero), PH1 of mixture spectrum; and form2’s proportion (α_2). The values in these fields are used to process the spectra according to the model equation included in the main text. This workflow mainly offers a visual assessment of the impact of

the provided values of the processing parameters and form2 proportion. No estimation is performed in this scenario.

- ***optimise only proportion***: This mode is identical to the previous one except that here the α_2 parameter is estimated from the data. For this purpose, we use an iterative optimisation algorithm. To initialise these computations, the value from the form2 proportion value [0-1] field is taken as the starting value for the underlying optimisation routine.
- ***optimise proportion and other processing parameters*** (DEFAULT choice): In this mode, the main parameter of interest α_2 is estimated jointly with the horizontal spectral shifts, and PH0 and PH1 of the mixture spectrum. The values specified in the corresponding input fields from *Spectral processing parameters (3)* section of the GUI are used as starting values (all zeroes by default if not changed otherwise by the user). If the *Initial PH0 from PepsNMR* box is ticked, the starting value of the PH0 parameter is estimated from the data using an external R package *PepsNMR* [4]. This option is advised while analysing not previously phase-corrected mixture spectra.

In the last two workflows, *only proportion* and *optimise proportion and other processing parameters*, once the optimisation algorithm reaches a solution, the found values are immediately displayed in the corresponding GUI input fields (*Spectral processing parameters (3)*). This feature enables running optimisation multiple times because the results obtained from the first optimisation call will be used as starting values for the subsequent round of optimisation, and so on. As a consequence, it becomes possible for the user to, for instance, quickly get automated estimation results (*optimise proportion and other processing parameters*), manually adjust the estimates in the relevant input fields, and re-estimate only the form2 proportion (*optimise only proportion*). Or any other combination of the described optimization modes is possible.

1.4 Spectral processing parameters (3)

Four characteristics of the analysed spectra are controlled by the input fields presented in Figure S5. Each input can be modified by entering a value from keyboard or clicking on an up- or down-arrow next to the field.

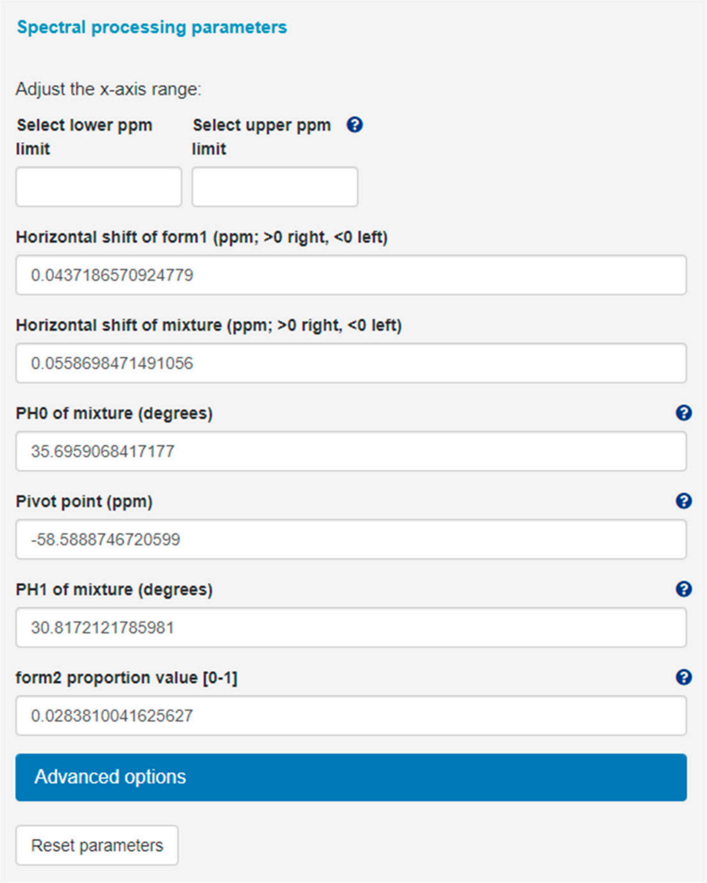

Figure S5 The input fields for spectral processing. Depending on the chosen optimisation mode, these fields can: 1) be directly applied to process and visualise the spectra, 2) be used as starting values for an iterative optimisation algorithm, 3) display the estimated model parameter values. The Reset parameters button restore the default values.

- **ppm range**: Set to invisible NULL values by default, resulting in analysis over the entire ppm range. The lower bound has to be less or equal than the upper bound.
- **chemical shifts**: These fields concern only the form1 template and mixture. Usually, small values (in ppm) suffice to align the three spectra horizontally. These parameters can be estimated automatically (see *Choose the optimisation mode (4)*).
- **phase correction:** These fields affect zero-order (PH0) and first-order (PH1) phase correction of the mixture spectrum (values in degrees). The pivot point (a point on the x-axis where PH1 correction will be zero) is automatically set to the index of the point corresponding to the largest peak in the mixture spectrum. Alternatively, the value of the pivot point can be manually supplied by the user by left-clicking on the desired region in the plot area. These parameters can be estimated automatically (see *Choose the optimisation mode (4)*), except the pivot point.
- **the form2 proportion α_2:** As described in the manuscript, α_2 (taking values from 0 to 1) is the model parameter corresponding to the "form2" template's contribution in the analysed mixture spectrum. Because of the imposed constraints, α_1 is equal to $1 - \alpha_2$. This parameter can be estimated (see *Choose the optimisation mode (4)*).

Lastly, at the bottom of the panel the *Reset parameters* button is located for restoring the input fields, including those hidden under *Advanced options*, to their default values. The parameter reset also occurs automatically whenever one of the input spectra is changed.

1.5 Advanced options (6)

Our Shiny app also offers several additional processing steps that are not optimised in the automated fitting routine (Figure S6).

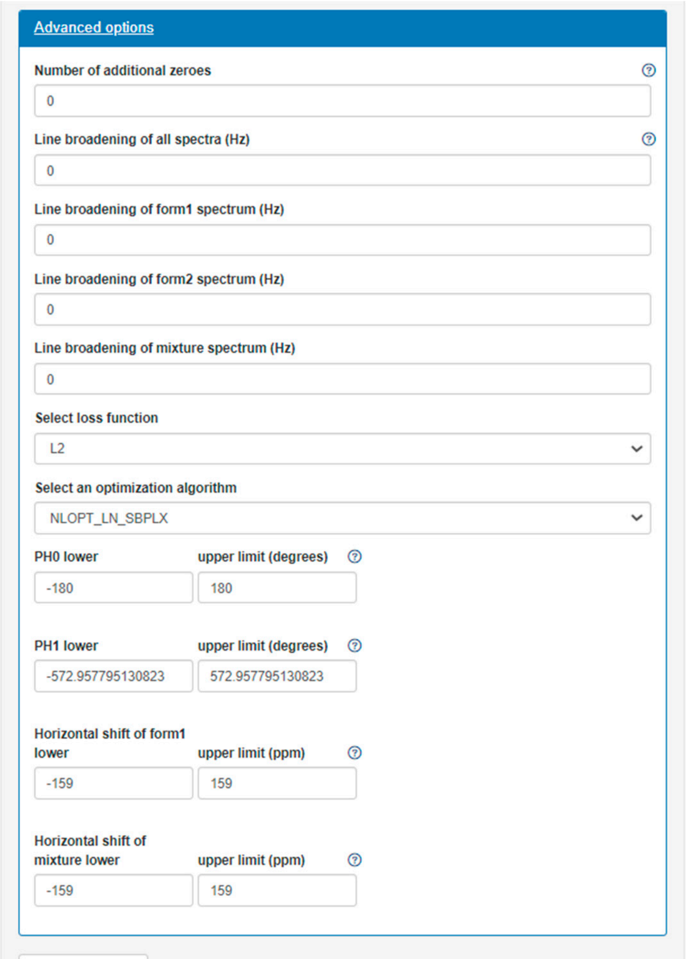

Figure S6 An overview of the advanced/additional options for spectral processing and iterative, constrained optimisation.

- **Number of additional zeroes (zero filling):** 0 by default. All three spectra are first inverse-Fourier transformed, appended with the specified number of points with zero intensity at the end of the FIDs, and Fourier transformed back to the frequency domain.
- **Line broadening (apodisation):** 0 by default, expressed in Hz. The lines in all three spectra at once or individually can be widened. Apodisation is implemented as follows:

  if GRPDLY parameter is set to zero, the spectrum is inverse-Fourier transformed, multiplied by a decaying exponential function: $\exp\left(-\frac{t \times LB \times \pi}{2 \times SW\_p}\right)$, where t=0,…, N-1 with N being the number of points in the spectrum (real part), *LB* the line broadening value from the input field, *SW_p* the spectral width of the spectrum (see *Load your own spectra (2)*), and finally Fourier-transformed to return to the frequency domain.

  If GRPDLY>0, the group delay correction is inversed (by rotating the spectrum as in phase correction), then the same process applies as when GRPDLY=0, and finally the group delay correction is applied on the spectrum. This procedure is to minimise the frowning/smiling effect at the ends of the spectrum.

- **Loss function:** either quadratic (L2 norm, by default) or absolute (L1 norm) deviation.
- **Optimisation algorithm:** NLOPT_LN_SBPLX by default. A selection of gradient-free optimisation algorithms offered by the *nloptr* function.
- **Lower and upper boundaries:** the *nloptr* function is suitable for solving non-linear, constrained optimisation problems. Thus, lower and upper boundaries must be specified when working in "optimise only proportion" and "optimise proportion and other processing parameters" estimation modes. Again, the default values are as follows: [-180, 180] degrees for PH0, [-572, 572] degrees for PH1, and [0,1] for α_2. For the horizontal shifts, the limits are automatically set to -/+ the half of the ppm axis range of all three spectra.

1.6 Fit visualisation (5)

The visualisation incorporated in the app's interface allows interactive exploration of the model fit. In particular, it displays: the original mixture spectrum, template spectra rescaled according to the model-estimated mixing proportions, the fitted line representing the linear combination of the template spectra, and the residual trace. Figure S7 shows the visualisation of the model fit results of exemplary data, and Figure S8 presents a zoomed view into the toolbar that appears in the top-right corner of the interactive graph. Below is a quick guidance on how to conveniently use the graph's interactive features based on the numbering from Figure S8:

- **Zooming in and out:** Keep the left-click and select the desired zoom region, then release the cursor. Buttons 4 or 5 are to be clicked for zooming in or out, or buttons 6 and 7 to restore the original axis ranges.
- **Moving around the plot region:** Simply press and keep left shift, then left-click and move the cursor to an another region.
- **Displaying only selected lines:** It is possible to turn off and on some lines shown in the graph by a left-click on the corresponding item in the graph's legend. In Figure S7, only the form1 and form2 spectra and the residual line are displayed.
- **Retaining zoom and legend item selection:** When the model is re-fitted and the visualisation recreated, the previously selected zoomed view or signal lines from the legend will be retained. Rapid double left-clicking on a legend item is not compatible with maintaining a zoomed view, and therefore double left-clicking is NOT recommended.
- **Taking a screenshot**: Clicking button 1 saves the current view.

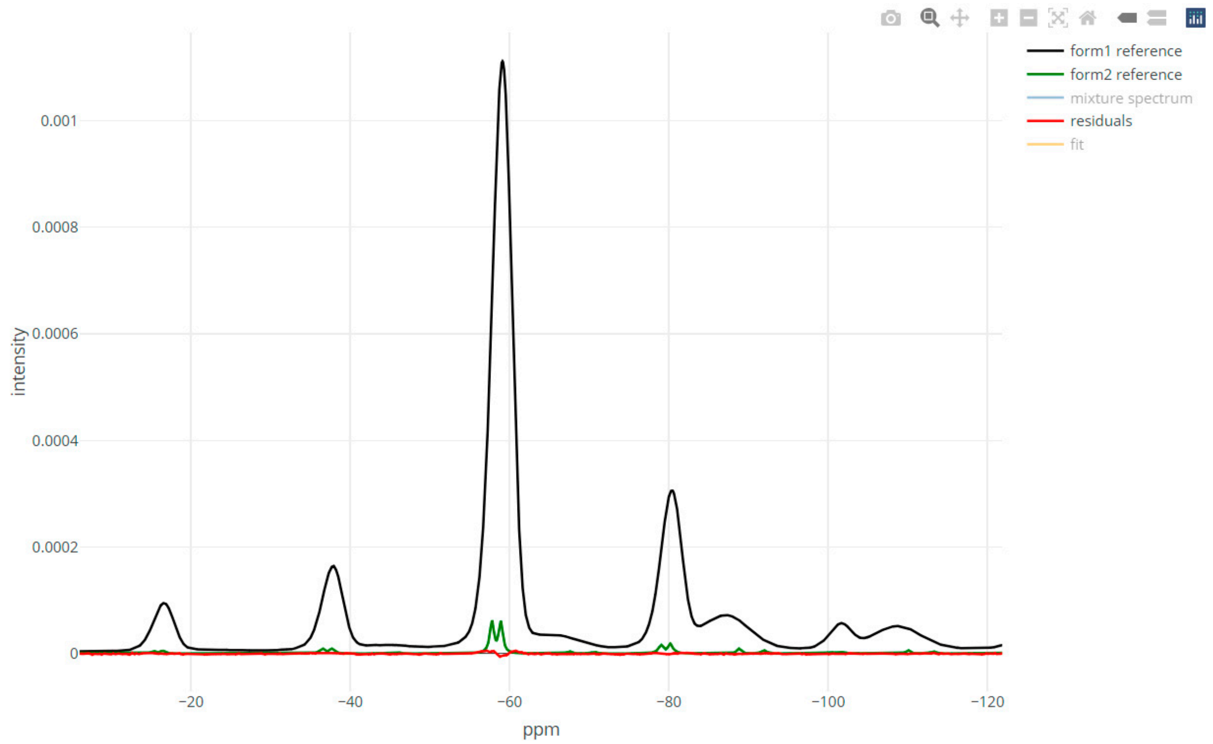

Figure S7 An Interactive visualisation (powered by R Plotly) of the optimisation results that allows the user to zoom in and out, move around the plot region, turn off and on spectral lines, and more.

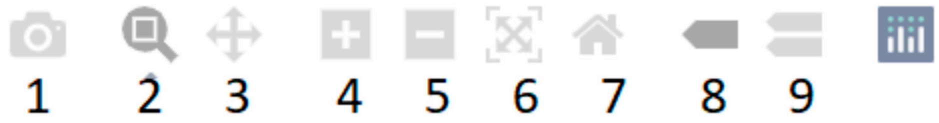

Figure S8 The toolbar enabling interactive exploration of the graph.

Do note that a similar visualisation (presenting raw spectra) is immediately displayed after loading input spectra.

1.7 Misc (7)

This section discusses several buttons that boost user experience and promotes the traceability of results (Figure S9).

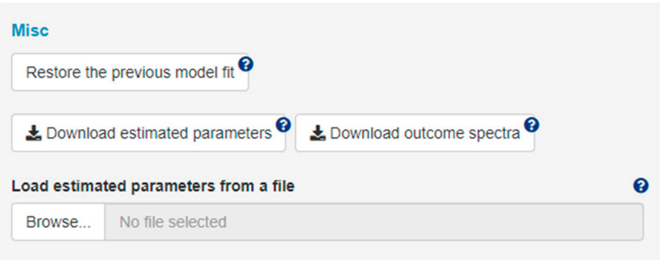

Figure S9 Various buttons facilitating undoing mistakes and traceability and reproducibility of analyses.

• **Restore the previous model fit:** if the fitting results turned out to be not satisfactory or the fitting routine has been run with invalid parameter values, click on *Restore the previous model fit* to restore the parameter values and press *Fit the model!* using the *no optimisation, apply the fixed processing values* estimation mode. This will undo the "mistake" and reproduce the "correct" graph.

• **Download estimated parameters:** Each time the *Fit the model!* button is pressed, a new row is created in the underlying CSV file that keeps track of the user actions, estimation results, and parameter settings. Columns included in that CSV file are listed and explained in Figure S10.
• **Download outcome spectra** After model fitting it is also possible to download the outcome spectral data used to create the interactive graph. The data is put into a CSV file.
• **Load estimated parameters from a file:** If the ongoing analysis has to be paused, download the CSV tracking file by clicking on *Download estimated parameters.* This results file can be uploaded again by clicking *Browse* under *Load estimated parameters from a file*. Although the CSV file may store multiple rows, only the last row will be loaded in, and its values will be propagated into the corresponding input fields in GUI. Then pressing *Fit the model!* using the *no optimisation, apply the fixed processing values* will reproduce the model fit graph.

Column	Description
`id`	Row identifier. Incremented by 1 if `Fit the model!` is pressed with the same set of spectra; if at least one spectrum is changed, `id` starts from 1.
`description_form1`, `description_form2`, `description_mix`	Spectral labels that come from the params input file.
`optim_algorithm`	Optimization algorithm name.
`fit_type`	The optimisation mode. Possible values: `no optimisation, apply the fixed processing values`, `optimise only proportion`, `optimise proportion and other processing parameters`.
`loss_function`	Quadratic (L2 norm; used by default) or Absolute deviation (L1 norm).
`mean_error_metric`	Either L2 or L1.
`prop_form2`	**Estimated form2 proportion** in [0, 1] interval.
`approve_fit`	A flag, possible values: empty string or `approved`.
`comment`	Comments provided by the user.
`ph0_mix,ph1_mix, pivot_point`	Phase correction parameters. Either estimated (`fit_type = optimise proportion and other processing parameters`) or manually provided by the user.
`ppm_form1,ppm_mix`	Chemical shifts of the form1 reference and mixture spectrum; either estimated (`fit_type = proportion and pre-processing parameters`) or manually provided by the user.
`add_zeroes, lb_global, lb_form1, lb_form2, lb_mix`	The number of appended zeroes to all three spectra, line broadening (in Hz) of all three spectra, additional line broadening (in Hz) of form1, form2, mixture spectra, respectively.
`*_lower, *_upper`	Lower and upper constraints of `ppm_form1, ppm_mix, ph0_mix, ph1_mix` required by the underlying optimization routine.
`ppm_range1 ppm_range2`	x-axis range used in the analysis; by default `NA` meaning no restrictions
`*_start`	Starting values of `prop_form2, ppm_amo, ppm_mix, ph0_mix, ph1_mix` required by the underlying optimization routine; by default 0, except `ph0_mix` if PepsNMR is applied.

Figure S10 The content of the underlying CSV tracking file (pressing Fit the model! creates a new row) that can be downloaded via Download estimated parameters.

Just under the model fit graph, a rectangle is positioned (Figure S11) that displays the estimated form 2 proportion, Mean Squared Error (if L2 loss function was selected) or Mean Absolute Deviation (in case of L1 loss function), initial and target spectrum size (these two numbers may differ due to zero filling). Additionally, when the model fitting is completed and the graph is drawn, the user can comment on the results by typing some text in a text field and saving

it in the tracking CSV file via the *Save the comments* button. The user can mark a good model fit by clicking on the *Approve the fit* button. This flag will also be saved into the underlying tracking CSV file and may be useful in distinguishing the good results from the bad ones.

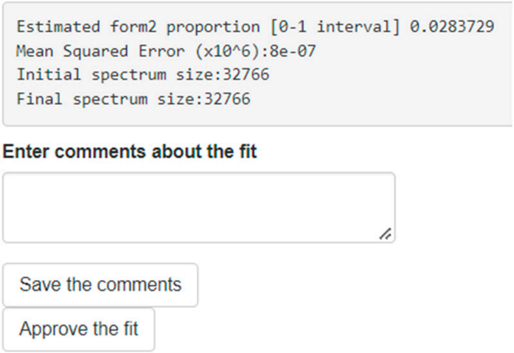

Figure S11 Information about the model fit is displayed underneath the interactive graph. Users can also comment and approve the optimisation results via a dedicated field and buttons.

References

1. R Core Team *R: A Language and Environment for Statistical Computing*; R Foundation for Statistical Computing: Vienna, Austria, 2022;
2. *Nloptr*;
3. Steven G. Johnson *The NLopt Nonlinear-Optimization Package*;
4. Martin, M.; Legat, B.; Leenders, J.; Vanwinsberghe, J.; Rousseau, R.; Boulanger, B.; Eilers, P.H.C.; De Tullio, P.; Govaerts, B. PepsNMR for 1H NMR Metabolomic Data Pre-Processing. *Analytica Chimica Acta* **2018**, *1019*, 1–13, doi:10.1016/j.aca.2018.02.067.
